# Comparison of the Effects of Lidocaine Prilocaine Cream (EMLA) and Lidocaine Injection on Reduction of Perineal Pain During Perineum Repair in Normal Vaginal Delivery 

**Published:** 2016-03

**Authors:** Roxana Kargar, Afsaneh Aghazadeh-Nainie, Hamid Reza Khoddami-Vishteh

**Affiliations:** 1 Department of Obstetrics & Gynecology, Shahid Beheshti University of Medical Sciences, Tehran, Iran; 2 Department of Internal Medicine, Shahid Beheshti University of Medical Sciences, Tehran, Iran

**Keywords:** Episiotomy, Pain, Lidocaine, Lidocaine Prilocaine Cream (EMLA)

## Abstract

**Objective:** To compare the efficacy of EMLA cream and lidocaine injection to reduce pain during episiotomy repair.

**Materials and methods:** A total of 46 primiparous women with normal pregnancy who referred for normal vaginal delivery and needed episiotomy repair were selected and randomly divided into two groups. For EMLA group, one hour before the estimated time of delivery, 5g of EMLA cream was applied to perinealmediolateral incision, and after the delivery of the fetus and placenta, again 5g of EMLA cream was applied to healthy skin around the episiotomy for repair. In the other group, lidocaine 2% was used before episiotomy and for its repair, too.

**Results:** Only 8 people (19%) were in need of further analgesia. The mean ± SD of pain during repair of episiotomy on the VAS scale in all cases was 4.2 ± 2.3 cm. Most people (97%) were satisfied with their episiotomy repair. Comparing the two groups of EMLA and lidocaine, there was no difference between the two groups in terms of the duration of episiotomy repair, need for further analgesia, pain on the VAS scale, and satisfaction with the repair method.

**Conclusion:** The findings of this study showed that the use of EMLA cream in the site of episiotomy incision in primiparous women can induce a level of analgesia equal to that of lidocaine, and cause a similar level of satisfaction.

## Introduction

Nowadays, different methods are applied to reduce pain during labor and the pain caused by episiotomy (1). Some of the commonly used methods include non-pharmaceutical methods such as hot packs (2, 3), cold water compresses (4), and massage of the perineum (5), and the use of local anesthetics (lidocaine gel or spray, lidocaine injection with or without vasoconstrictor) (6, 7).Although there is no general agreement on the identification of one or more of the methods as the main methods, the most commonly used method is the injection of topical anesthetic. On the other hand, some other medical specialties have reported that the use of topical products such as sprays (8), gels (9, 10), and creams/ointments (11, 12) are good alternatives to injectable anesthetics. The benefits of using topical products include the following: having a local effect without significant systemic absorption, ease of use, and the option to be used by the patient (13).

EMLA cream (Eutectic Mixture of Local Analgesics) is one of the local anesthetics. This cream is a combination of two analgesics equally combined including lidocaine 2.5% and prilocaine 2.5%. The cream is applied on the intact skin under a patch and causes skin numbness via the release of lidocaine prilocaine from the cream to the epidermis and dermis layers, and it makes an effect on pain receptors in the skin and nerve endings. The onset, depth, and duration of anesthesia caused by the cream depends on the duration of cream use; for some procedures like intravenous catheters and venipuncture the cream must remain under the patch for at least one hour. The desired effect was achieved one hour and the maximum effect was achieved two to three hours after the use of the cream and its effect has remained one to two hours after cleaning the skin. A faster absorption of about five to 10 minutes occurred in genital mucosa. After five to 10 minutes of using EMLA cream on women’s genital mucosa, the mean duration of anesthesia for laser was 15 to 20 minutes. The amount of lidocaine prilocaine of EMLA cream which is systemically absorbed is directly dependent on the duration of use and the area under the cream (14). This cream has trivial side effects, which include tingling, coldness and warmth of the skin, pallor or redness of the skin, and swelling. Allergic or systemic reactions of the skin (rash or hives) are among the rare side effects.

EMLA Cream is used widely in small pediatric (15, 16), dermatology (17), and surgeries (18). This combination is used for a number of small operations in women, including small surgeries of genital mucosa, genital warts, vulva biopsy, laser treatment for CIN lesions, and hysteroscopy (19). Studies conducted on the local anesthetics combinations used in obstetrics operations are more focused on pain during second stage of labor (7) or pain in the early postpartum period (20, 21). Few studies have focused on pain during perineal repair caused by episiotomy (13). Hence this study was aimed to compare the effects of lidocaine prilocaine cream (EMLA) and lidocaine injection on reduction of perineal pain during episiotomy repair in normal vaginal delivery.

## Materials and methods

This randomized clinical trial was conducted on primiparous women who were admitted to Shohada-e-Tajrish Hospital for normal vaginal delivery in 2011. The inclusion criteria were: gestational age more than 37 weeks, normal vaginal delivery, single pregnancy, and cephalic presentation. The exclusion criteria at the beginning of the study were as follow: multiparity, sensitivity to topical anesthetics or lidocaine, need to epidural anesthesia, heart diseases, uncontrolled diabetes mellitus, neurological diseases which affect lower limb, and use systemic or inhalation opium to reduce pain during labor. The exclusion criteria during the study were: using forceps for delivery, emergency cesarean, cervical tears, and the presence of several types of rupture. Based on the only study conducted via the same method by Franchi et al. (13) and considering a reduction of 20% in samples due to emergency cesarean and using forceps for delivery, the sample size for this study was estimated 23 patients in each group (a total of 46 patients). Samples were randomly placed in one of the two groups of lidocaine and EMLA. However, at the end, a total of 43 patients remained in the study (three persons in EMLA group were excluded during the study because of the exclusion criteria). The study was ethically approved by ShahidBeheshti University of Medical Sciences with code of 268 at 2011-09-23 and it was registered in the website of clinical trials of Iran (www.irct.ir) with a registration number “IRCT2014100219373N1”.

After explaining the procedures of the project, written informed consent was obtained from patients and their basic demographic information such as age, height, weight, gestational age, and cervical dilation were recorded. Then, the patients in need of repair of episiotomy were allocated to intervention and control groups. Episiotomy was performed in all patients via mediolateral method. To reduce pain lidocaine prilocaine cream (EMLA) was used for the intervention group, and lidocaine was injected for the control group. EMLA cream is a pharmaceutical product manufactured by Tehran Shimi pharmaceutical; it is a 30g topical cream which is packed in 5g sterile tubes. Lidocaine hydrochloride 2% manufactured by Rasht Caspian Company was used for injection. In the intervention group, one hour before the estimated delivery time, 5g of EMLA cream was applied at a 9cm dilatation of cervix to the mediolateral incision site in the perineum. Since sodium hydroxide which is a component of the cream irritates the baby's eyes, to avoid contact with the head of the newborn, the remaining of the cream was cleared before delivery. To reduce the pain of episiotomy, no other method of anesthetics was used. After delivery of the fetus and placenta, 5g of EMLA cream was applied to the healthy skin surrounding the episiotomy site and repair was performed after 10 minutes. If there was active bleeding during this period, packing method was used. In the control group, 5cc of lidocaine 2% was injected before episiotomy in the site of mediolateral episiotomy incision. Then, after the delivery of fetus and placenta postpartum, 5cc of lidocaine 2% was injected into line of episiotomy incision and the repair was performed after 10 minutes. Repair of episiotomy was performed by a trained first-year resident and similar suture threads and repair technique were applied. If more anesthetic was needed during repair, lidocaine 2% was used in both groups.

Before leaving the delivery room (about two hours after delivery), delivery characteristics of patients (including the presence or absence of labor induction, the duration of the first to the third stages of delivery, type of placenta delivery, and the newborn weight) were recorded; in addition, the patients were asked to rate their pain during repair of episiotomy on a VAS scale (10 cm vertical linear scale in which zero indicates no pain and 10 indicates maximum pain). The rate of patients’ satisfaction with repair method was determined using a Likert scale question with five options (very satisfied, satisfied, neither satisfied nor dissatisfied, dissatisfied, very dissatisfied). The extra amount of lidocaine use was also recorded. Same antibiotic regimen was used for all patients for prevention of wound infection. Wound infection symptoms such as pain, suppuration, fever, vulva edematous, and red and swollen mucosa were described for patients at discharge, and they were advised to refer to physicians in case of the presence of any symptoms. In a visit, one week after the delivery, the episiotomy site was examined for infection.

Data analysis was performed using SPSS 13 statistical software. The qualitative variables were described using frequency and relative frequency (%) and quantitative variables were described using range, mean, and standard deviation. Chi-square test was used to compare qualitative variables between the two groups and patients’ satisfaction with the methods; independent t-test was also used to compare the quantitative variables and the severity of pain between the two groups. P value <0.05 was considered as the significance level.

## Results

Range and mean ± SD age of women were 17-37 years and 24 ± 4 years, respectively. The two groups were not significantly different in terms of age, weight, height, and BMI (Table 1). Range and mean ± SD gestational age of the subjects were 35-42 weeks and 39 ± 1 weeks, respectively. A total of 13 patients (30%) underwent induction. Range and mean ± SD cervical dilation were 0–8 cm and 2.3 ± 1.8cm, respectively. The delivery of placenta occurred spontaneously in all women. Moreover, mean ± SD birth weight in all subjects was 3250 ± 407g. There were no significant differences between the two groups in terms of gestational age, the induction of labor, cervical dilation, length of different stages of delivery, and birth weight (Table 2).

Mean ± SD duration of keeping EMLA cream on the site of episiotomy incision before delivery in EMLA group was 53 ± 21 minutes. The mean ± SD duration of episiotomy repair in all subjects was 26 ± 10 minutes. Only eight patients (19%) were in need of more analgesia; 5cc of lidocaine 2% was used for these cases. Range and mean ± SD pain during episiotomy repair on a VAS scale were 1–8 cm and 4.2 ± 2.3 cm, respectively. Of all, 40 patients (93%) were partially or fully satisfied with their episiotomy repair. Table 3 shows the characteristics of episiotomy, its repair, and the differences in patients’ satisfaction with the repair method between the two groups. Episiotomy repair duration was not significantly differ between EMLA group and lidocaine group (26 ± 11 min *vs.*25 ± 9 min, respectively, p = 0.890). 

**Table 1:** Comparison of demographic characteristics between lidocaine and EMLA groups

	**EMLA group** **mean ± SD**	**Lidocaine group** **mean ± SD**	**p value** *
Age, year	23 ± 4	25 ± 9	0.397
Height, cm	158 ± 7	160 ± 7	0.432
Weight, Kg	66 ± 8	72 ± 11	0.087
BMI	26.6 ± 3	28.1 ± 3.7	0.167

* Independent sample t test

**Table 2:** Comparison of pregnancy characteristics between lidocaine and EMLA groups

	**EMLA group**	**Lidocaine group**	**p value**
Gestational age mean ± SD	39 ± 1	39 ± 2	0.685*
Delivery induction Frequency (%)	6 (30%)	7 (30%)	0.975**
Cervix dilatation, cm mean ± SD	2.2 ± 1.5	2.5 ± 2	0.648*
Duration 1^st^ stage of labor, hour mean ± SD	9 ± 9	9 ± 6	0.714*
Duration 2^nd^ stage of labor, min mean ± SD	32 ± 18	35 ± 17	0.565*
Duration 3^rd^ stage of labor, min mean ± SD	5 ± 1	5 ± 1	1.000*
Newborn weight, gram mean ± SD	3217 ± 339	3279 ± 464	0.167*

* Independent sample t test,

** Chi square test

**Table 3:** Comparison of episiotomy and its repair characteristics and satisfaction from repair between lidocaine and EMLA groups

		**EMLA group**	**Lidocaine group**	**P value**
Episiotomy repair duration, min mean ± SD		26 ± 11	25 ± 9	0.890*
need of further analgesia Frequency (%)		3 (15%)	5 (22%)	0.571**
Pain during repair, VAS mean ± SD		4.1 ± 2.5	4.3 ± 2.2	0.730*
Satisfaction Frequency (%)	neither satisfied nor dissatisfied	1 (5%)	2 (9%)	0.681**
	Partial satisfied	13 (65%)	12 (52%)	
	Fully satisfied	6 (30%)	9 (39%)	

* Independent sample t test,

** Chi square test

Three persons (15%) of EMLA group and 5 persons (22%) lidocaine group needed further analgesia (p = 0.730). In addition, 19 persons (95%) of EMLA group and 21 persons (91%) partially or fully satisfied from their episiotomy repair which has not significantly different (p = 0.681).

Moreover, nine patients did not refer for the visit which was intended to check episiotomy infection one week later; the other 34 patients did not have the symptoms of wound infection at the incision site.

## Discussion

Although several studies have been conducted on the effects of EMLA cream in a variety of gynecologic and delivery procedures, to the best of our knowledge there has been only one study so far comparing the effect of using topical EMLA cream with the injection of topical anesthetic to reduce the pain caused by the perineal sutures after childbirth; accordingly, our study is the second study in this field. The mentioned study was conducted by Franchi et al. in 2009 and compared the effect of EMLA cream with that of mepivacaine injection used for perineal tears and episiotomy. The results of the study showed that EMLA group had lower pain scores compared with the other group. The proportion of women who needed more anesthetic were similar in both groups, however, a larger proportion of women in the EMLA group had expressed satisfaction with the technique used for analgesia (83.8% in EMLA group, 53.3% in mepivacaine group). Finally, it was stated that EMLA cream was an appropriate and effective alternative for intravenous injections to reduce pain during perineal repair (13). Although our study did not show the superiority of EMLA cream on lidocaine in terms of pain reduction and women's satisfaction, our results indicated that EMLA cream is at least as effective as lidocaine injecting and makes similar level of satisfaction. Hence EMLA can be an alternative for lidocaine which has been routinely used for years to repair perineal tears and episiotomy. Consequently, it can be said that the results of our study can complete the results of Franchi et al.’s study (13). On the other hand, since the costs of manufacturing EMLA cream and lidocaine are approximately equal, it is likely that further studies about perineal tears with larger sample size will lead to some significant results.

Apart from repairing the perineum during childbirth, the EMLA cream has also been used in other cases and surgical procedures for women. It is also used for small surgeries of genital mucosa (22), controlling the pain of cryotherapy in the treatment of papilloma virus (23), removing genital warts, vulva biopsy, laser treatment for CIN lesions, and hysteroscopy (19), hysterosalpingography (24). Finally, it has been stated that EMLA cream can be an efficient alternative to the injectable analgesics used for local obstetric and gynecologic procedures.

## Conclusion

The findings of this study showed that the use of EMLA cream in the site of episiotomy incision (from one hour before the delivery and then before episiotomy repair) in primiparous women with normal vaginal delivery can induce a level of analgesia equal to that of lidocaine, and cause a similar level of satisfaction.
